# Revealing critical mechanisms in determining sorghum resistance to drought and salt using mRNA, small RNA and degradome sequencing

**DOI:** 10.1186/s12870-024-05230-1

**Published:** 2024-06-13

**Authors:** Qiong Li, Jibin Wang, Qian Liu, Junhan Zhang, Xinlei Zhu, Yinpeng Hua, Ting Zhou, Songxian Yan

**Affiliations:** 1 Department of Brewing Engineering, Moutai Institute, Renhuai, 564507 Guizhou China; 2 Department of Resources and Environment, Moutai Institute, Renhuai, 564507 Guizhou China; 3 School of Life Sciences, Zhengzhou University, Zhengzhou, 450001 China; 4 School of Agricultural Sciences, Zhengzhou University, Zhengzhou, 450001 China

**Keywords:** Sorghum, Transcription factor, Drought, Salt, Multiomics

## Abstract

**Background:**

Plant growth and development are severely threatened by drought and salt stresses. Compared with structural genes, transcription factors (TFs) play more pivotal roles in plant growth and stress adaptation. However, the underlying mechanisms of sorghum adapting to drought and salt are insufficient, and systematic analysis of TFs in response to the above stresses is lacking.

**Results:**

In this study, TFs were identified in sorghum and model plants (*Arabidopsis thaliana* and rice), and gene number and conserved domain were compared between sorghum and model plants. According to syntenic analysis, the expansion of sorghum and rice TFs may be due to whole-genome duplications. Between sorghum and model plants TFs, specific conserved domains were identified and they may be related to functional diversification of TFs. Forty-five key genes in sorghum, including four TFs, were likely responsible for drought adaption based on differently expression analysis. MiR5072 and its target gene (*Sobic.001G449600*) may refer to the determination of sorghum drought resistance according to small RNA and degradome analysis. Six genes were associated with drought adaptation of sorghum based on weighted gene co-expression network analysis (WGCNA). Similarly, the core genes in response to salt were also characterized using the above methods. Finally, 15 candidate genes, particularly two TFs (Sobic.004G300300, HD-ZIP; Sobic.003G244100, bZIP), involved in combined drought and salt resistance of sorghum were identified.

**Conclusions:**

In summary, the findings in this study help clarify the molecular mechanisms of sorghum responding to drought and salt. We identified candidate genes and provide important genetic resource for potential development of drought-tolerant and salt-tolerant sorghum plants.

**Supplementary Information:**

The online version contains supplementary material available at 10.1186/s12870-024-05230-1.

## Background

The ongoing global issues of drought and soil salinization are considered significant stress factors that constrain agricultural production [1–5]. All over the world, food security is challenged by multiple factors such as rapidly increasing food demand, scarce freshwater resources, and continuous incensement of saline and alkaline land [6–8]. Approximately 43% of the world’s cultivated land area is affected by arid and semi-arid climates [9, 10]. In the world, over 1 billion ha lands are under the threat of salinity, and about 30% of arable lands are being affected by salinity in China [11, 12]. In addition, drought and salt stresses often occur together, leading to the combined stress on plant growth. According to relevant studies [13, 14], among various stresses, combined salt and drought stress can commonly lead to an over 40% reduction in crop yield. Therefore, increasing attention should be paid to the effect of drought, salinity and their combination on plant growth and development.

In the semi-arid tropical and sub-tropical fields where drought and salt often co-occur [15–17], sorghum (*sorghum bicolor* (L.) Moench) is wildly grown for its stress-adaptive traits, including high water-use efficiency, salinity tolerance, alkalinity tolerance and C4 photosynthesis [18]. Sorghum may be one of the best crop plants to study their resistance to drought or salt and even their combination. Plants adapt to single or multiple environmental stresses by regulating gene transcription, usually [19]. MicroRNA(miRNA)-controlled post transcriptional gene regulation is also demonstrated to be important for the adaption of plants to stresses. Small RNA and mRNA transcriptomes have been used to identify the expression profiles of miRNAs and genes in response to drought and salt in sorghum [20–27]. However, the molecular regulatory mechanisms of sorghum in response to drought and salt are not very clear, especially the regulatory process involving microRNAs (miRNAs) and their target genes.

In eukaryotic organisms, the process of transcription initiation is highly complex and often requires the assistance of multiple transcription factors (TFs) [28, 29]. TFs are the proteins that located in cell nucleus and interact specifically with *cis*-acting elements in genes promoter regions, and they regulate gene transcription with specific strength at specific times and locations. TFs generally form complex with RNA polymerase II to participate in the transcription initiation of genes [30, 31]. TFs usually take part in plants growth, development, secondary metabolism, and stress resistance by controlling a great many genes, thereby they may be better candidate genes for improving agronomic traits and cultivating new varieties in crops [16, 32].

Currently, the molecular regulatory mechanisms of sorghum in response to drought and salt stress are being revealed, while miRNAs-genes regulatory module about drought and salt stress, and the adaptive mechanisms of sorghum in response to combined drought-salt stress are not very clear. In addition, the functions of TFs in regulating drought and salt stress resistance were not systematically understood. In this study, a comprehensive study of TFs in sorghum, *Arabidopsis thaliana* and rice was conducted. The conserved domains of TFs were compared between sorghum and model plants (*Arabidopsis thaliana* and rice), and the syntenies among these species were performed. The responses of miRNAs, genes and TFs to drought and salt were explored in sorghum using small RNA, mRNA and degradome sequencing. Potential candidate miRNAs, genes and TFs involved in drought, salt, and their combination were identified. Here, important clues for underlying the molecular basis of sorghum adapting to drought and salt will be provided.

## Results

### Identification, conserved domain, and synteny analysis of TFs

There were 1859, 1717 and 1862 TFs in sorghum, *Arabidopsis thaliana* and rice, respectively (Fig. S1). The number of TFs between sorghum and rice was basically consistent, while TFs in *Arabidopsis thaliana* were less than the above species (Fig. S1). The distribution of sorghum genes and TFs on chromosomes was identified. We found that genes and TFs were mainly located on two ends of chromosomes (Fig. S2). Chromosome 01, 02, and 03 contained more TFs than the other chromosomes, while a peak of TFs quantity occurred on the end of chromosome 05 (Fig. S2).

Various conserved domains were found in sorghum and model plants (*Arabidopsis thaliana* and rice). In sorghum and model plants, most conserved domains were consistent (Table S1). However, several distinct domains were identified in sorghum and model plants. For example, B3_DNA, PB1 and PHA03247 domains were specific in model plants ARFs ( a type of TFs), and sorghum ARFs specifically contained PHA03379; Compared with sorghum, PLN02705 and PLN02905 domains were only identified in model plants TFs; And PTZ00449 domain was in sorghum TFs but not in model plants TFs. The matters need attention are that some conserved domains were only presented in model plants TFs, and no domains were identified from sorghum TFs. For example, there were Bbox1_BBX-like, Bbox_SF and BBOX domains in model plants DBBs (a type of TFs), but sorghum DBBs contained no domains; DELLA and GRAS domains were in model plants GRAS TFs, while there was no domain in sorghum ones. Something else interesting was that two types of TFs (ARR-B and VOZ) shared no domains in both model plants and sorghum.

Duplication events within gene pairs were identified in duplicated blocks of sorghum, *Arabidopsis thaliana* and rice genomes, and 447, 503 and 400 gene pairs were respectively in the above plant species (Fig. S1). We performed collinearity analysis between sorghum and model plant species. There were 300 and 2010 TF pairs were identified in sorghum-*A. thaliana* and sorghum-rice, respectively (Fig. S1). To clarify divergence among these gene pairs, the non-synonymous to synonymous substitutions (Ka/Ks) ratios were identified. The Ka/Ks ratios of all TF pairs in sorghum were less than 1, while the Ka/Ks ratio of Sobic.003G246800-Sobic.009G243600 was 1.005141 (Table S2). The Ka/Ks ratios of *Arabidopsis* TF in pairs ranged from 0.046 to 0.509 (Table S3). All rice TF pairs shared Ka/Ks ratios with less than 1 (Table S4). The Ka/Ks ratios between all TF pairs in sorghum-*A. thaliana* were all less than 0.5 (Table S5). There were 6 sorghum-rice TF pairs (Sobic.002G280800-LOC_Os09g36910, Sobic.007G176700-LOC_Os09g36910, Sobic.003G253200-LOC_Os01g55340, Sobic.006G118200-LOC_Os04g39960, Sobic.007G156700-LOC_Os02g52670 and Sobic.008G073400-LOC_OS14g10660) sharing Ka/Ks ratios over 1, and Ka/Ks ratios of the other TF pairs were less than 1 (Table S6).

### Identification of DEGs in response to drought stress at drought-resistant and drought-sensitive sorghum genotypes

**Fig. 1:**
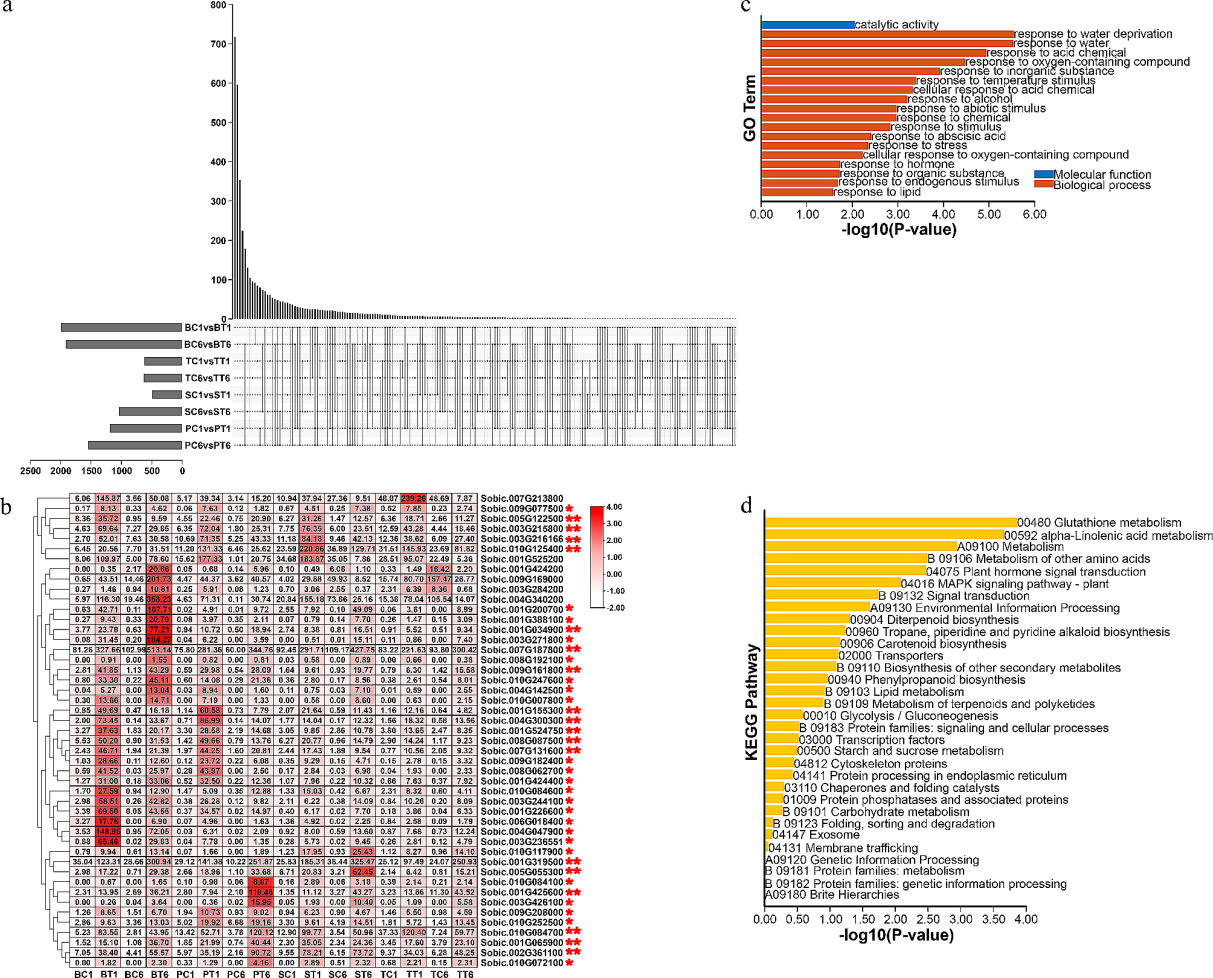
The DEGs between control and drought-treated sorghum seedlings from different genotypes (**a)** Venn diagram showing the common DEGs of the eight pairwise comparisons. (**b**) Expression profile of the common DEGs in the eight pairwise comparisons. Genes with high expression which induced by drought were labeled with red asterisk. (**c**) GO analysis of the common DEGs. (**d**) KEGG analysis of the common DEGs

After PEG treatment for 1 and 6 h, the differentially expressed genes (DEGs) that passed the cut-off |Log_2_FC| > 1 and q-value < 0.05 were identified in two drought-resistant sorghums (BTx623 and SC56) and two drought-sensitive sorghums (Tx-7000 and PI-482,662) (Table S7-S14). Forty-seven DEGs were differentially expressed at both 1 and 6 h in all for sorghum genotypes, and 3 of them were TFs (Fig. 1a). A total of 41 genes were commonly induced by drought, and 18 DEGs (*Sobic.005G122500*, *Sobic.003G215800*, *Sobic.003G216166*, *Sobic.010G125400*, *Sobic.001G034900*, *Sobic.007G187800*, *Sobic.009G161800*, *Sobic.001G155300*, *Sobic.004G300300*, *Sobic.001G524750*, *Sobic.008G087500*, *Sobic.007G131600*, *Sobic.001G319500*, *Sobic.005G055300*, *Sobic.001G425600*, *Sobic.010G084700*, *Sobic.001G065900* and *Sobic.002G361100*) showed relatively high expression level (Fig. 1b). To identify the function of DEGs, GO and KEGG enrichments were performed (Fig. 1c and d). These DEGs were involved in response to water, temperature, abiotic stimulus, abscisic acid, stress and hormone signals based on GO analysis (Fig. 1c). According to KEGG analysis, secondary metabolites, carbohydrate and energy metabolism (i.e., carotenoid biosynthesis, biosynthesis of other secondary metabolites, glycolysis, starch and sucrose metabolism, and carbohydrate metabolism) were identified (Fig. 1d).

**Fig. 2:**
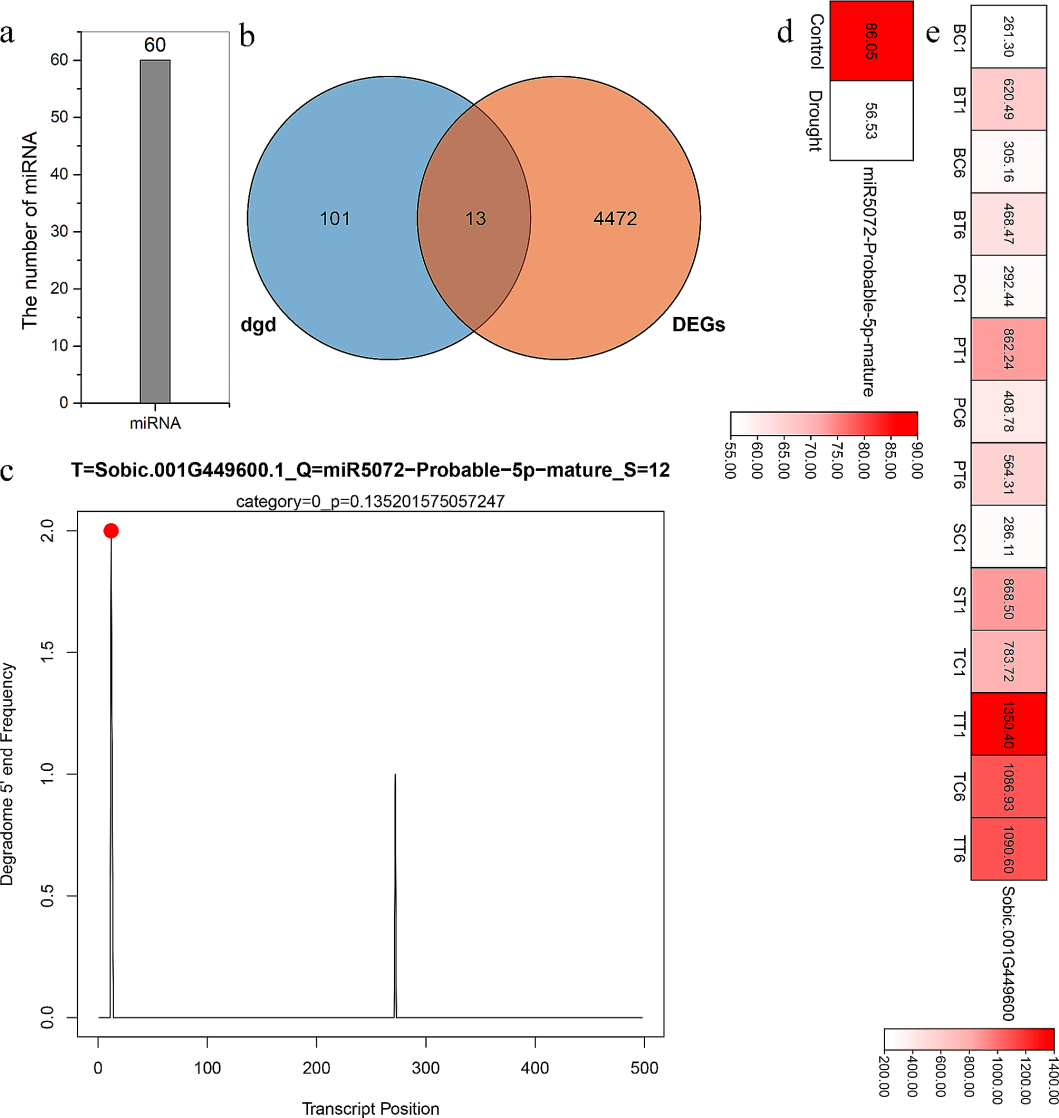
The analysis of miRNA and its target responding to drought. (**a**) The number of miRNAs identified in control and drought-treated sorghum. (**b**) Venn diagram showing the common genes between the targets of miRNAs identified by degradome sequencing and DEGs in response to drought stress. (**c**) Target plot (t-plot) for miR5072 targets confirmed by degradome sequencing. (**d**) Expression analysis of miR5072 in response to drought. (**e**) Expression analysis of miR5072’ target (*Sobic.001G449600*) in response to drought

Usually, microRNAs (miRNAs) control plant growth and stress responses through their target genes. MiRNAs and their targets share opposite expression patterns, generally [33]. There were 60 miRNAs involving in the adaption of sorghum to drought (Fig. 2a and Table S15). And their targets were identified using degradome sequencing (Table S16-17). Among the above target genes, 13 of them were the DEGs which identified in Table S7-S14 (Fig. 2b). According to the degradome analysis, miR5072-probable-5p-mature was predicted to bind to 12 bp at 5’ end of the *Sobic.001G449600.1* mRNA, and the binding site was confirmed by the target plot of miR5072-probable-5p-mature (Fig. 2c). The expression of miR5072-probable-5p-mature was repressed after drought treatment (Fig. 2d), while its target was up-regulated in BTx623, Tx-7000 and PI-482,662, and at 1 h in SC56 (Fig. 2e).

**Fig. 3:**
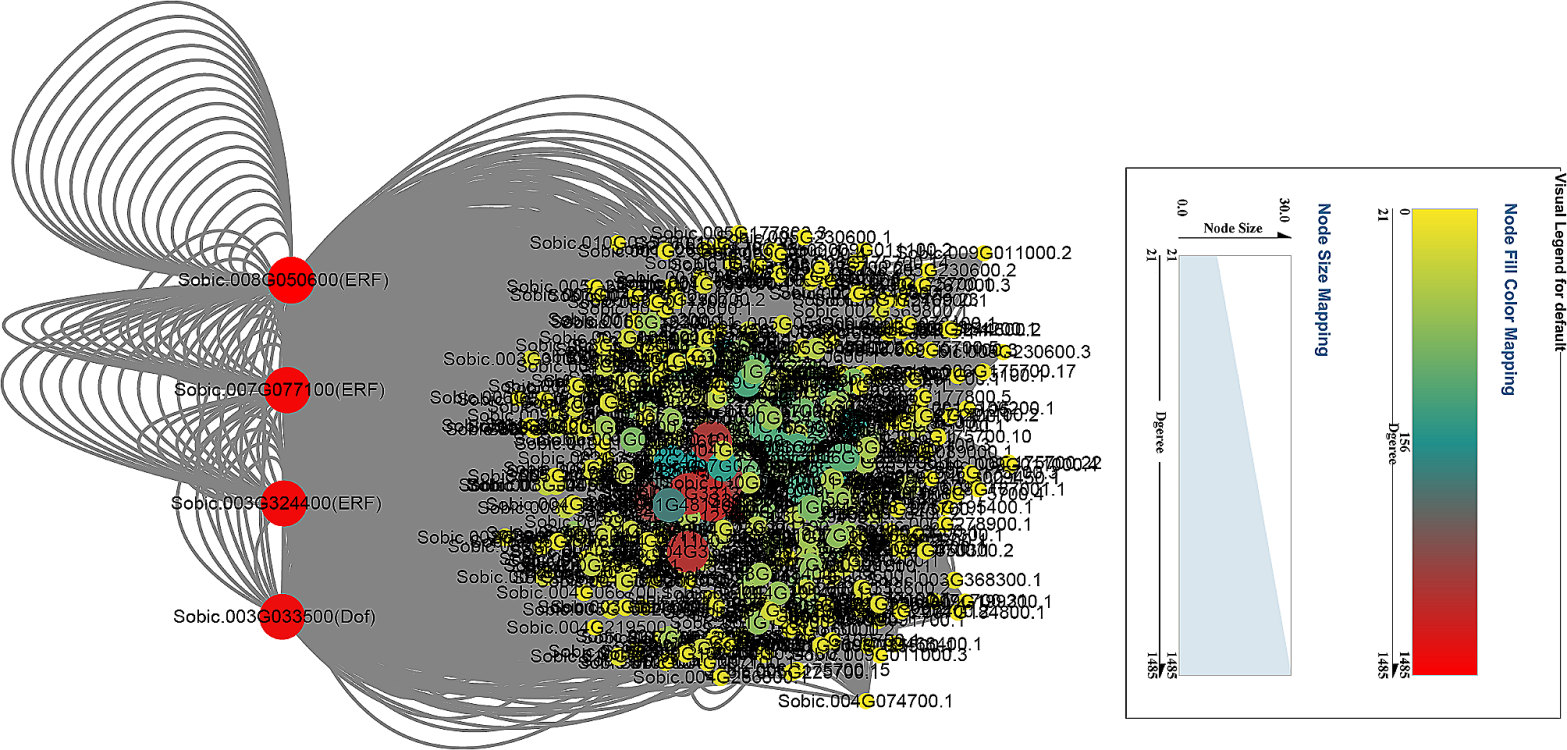
Regulatory network of TFs-mediated drought response in sorghum

Transcription factors (TFs) serve as essential switches of regulatory cascades in many plant processes, including developmental and metabolic processes, biotic and abiotic stresses [34]. In order to identify TFs regulating drought adaption, the TFs-DEGs network was built with all DEGs (Fig. 3). Four TFs, Sobic.008G050600 (ERF), Sobic.007G077100 (ERF), Sobic.003G324400 (ERF) and Sobic.003G033500 (Dof), were likely to be candidate genes for sorghum resistance.

### Identification of WGCNA modules and hub genes associated with drought stress

**Fig. 4:**
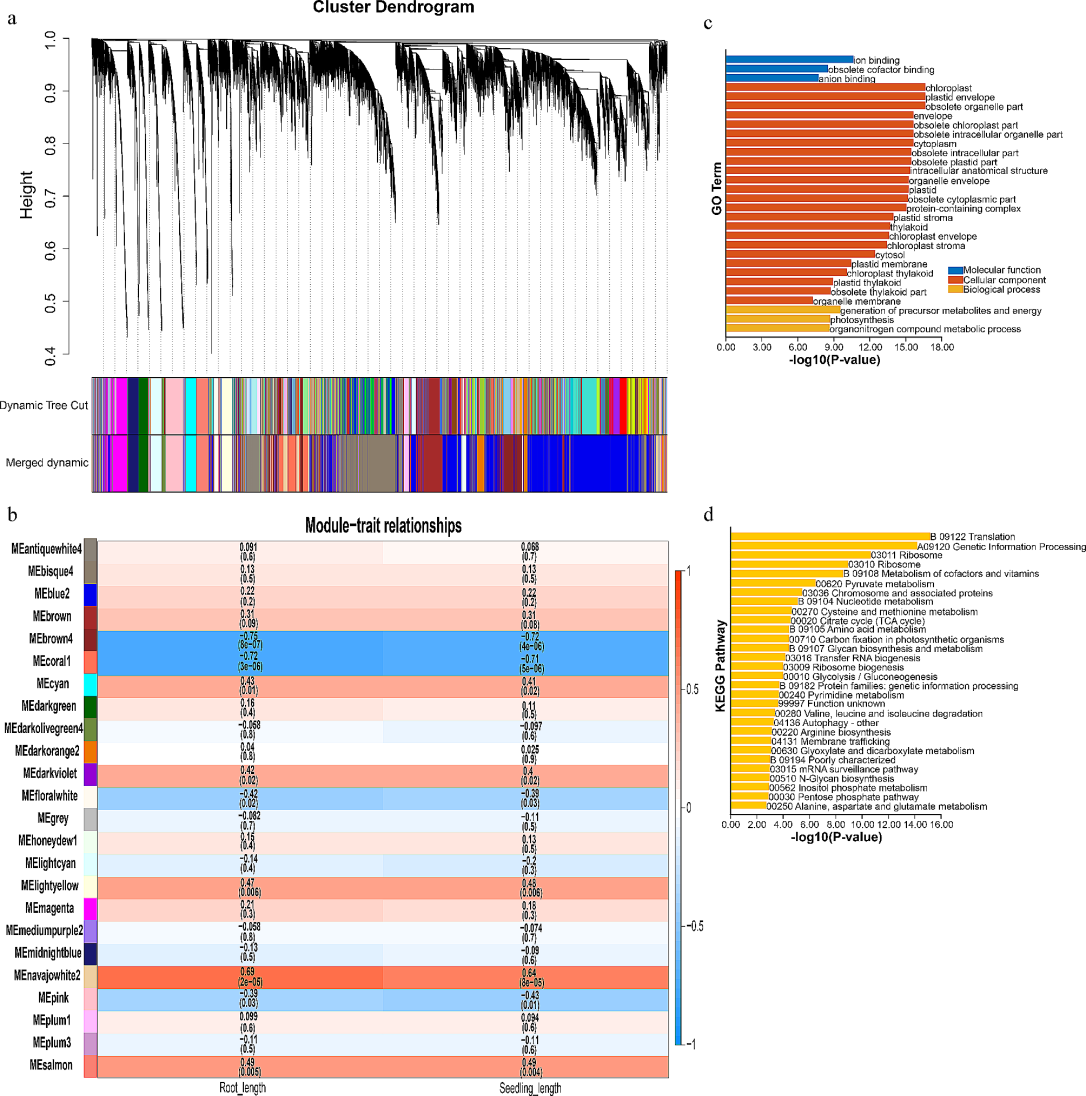
WGCNA of gene expression and root as well seedlings length in sorghum under drought stress. (**a**) Hierarchical clustering tree showing 24 modules of co-expressed genes by WGCNA. (**b**) The correlations between modules and sorghum growth. The number in each cell indicates the correlation coefficient (*r*), and the *P*-value (in parentheses) represents correlation significance (*P* < 0.05 indicated the significant correlation). (**c**) GO analysis of the genes in ‘brown4’, ‘coral1’ and ‘navajowhite2’ modules. (**d**) KEGG analysis of the genes in ‘brown4’, ‘coral1’ and ‘navajowhite2’ modules

In WGCNA, twenty-four modules were identified to associate with phenotypes using 21,204 expressed genes (Fig. 4a). Module-trait relationship analysis revealed that root and seedling length were negatively correlated with ‘brown4’ (*r* = -0.75, *p* < 0.05; *r* = -0.72, *p* < 0.05) and ‘coral1’ (*r* = -0.72, *p* < 0.05; *r* = -0.71, *p* < 0.05) modules, and positively correlated with ‘navajowhite2’ (*r* = 0.69, *p* < 0.05; *r* = 0.64, *p* < 0.05) module (Fig. 4b). The genes in the above modules were significantly enriched in energy metabolism, such as generation of precursor metabolites and energy, photosynthesis, TCA cycle, carbon biosynthesis and glycolysis (Fig. 4c and d).

**Fig. 5:**
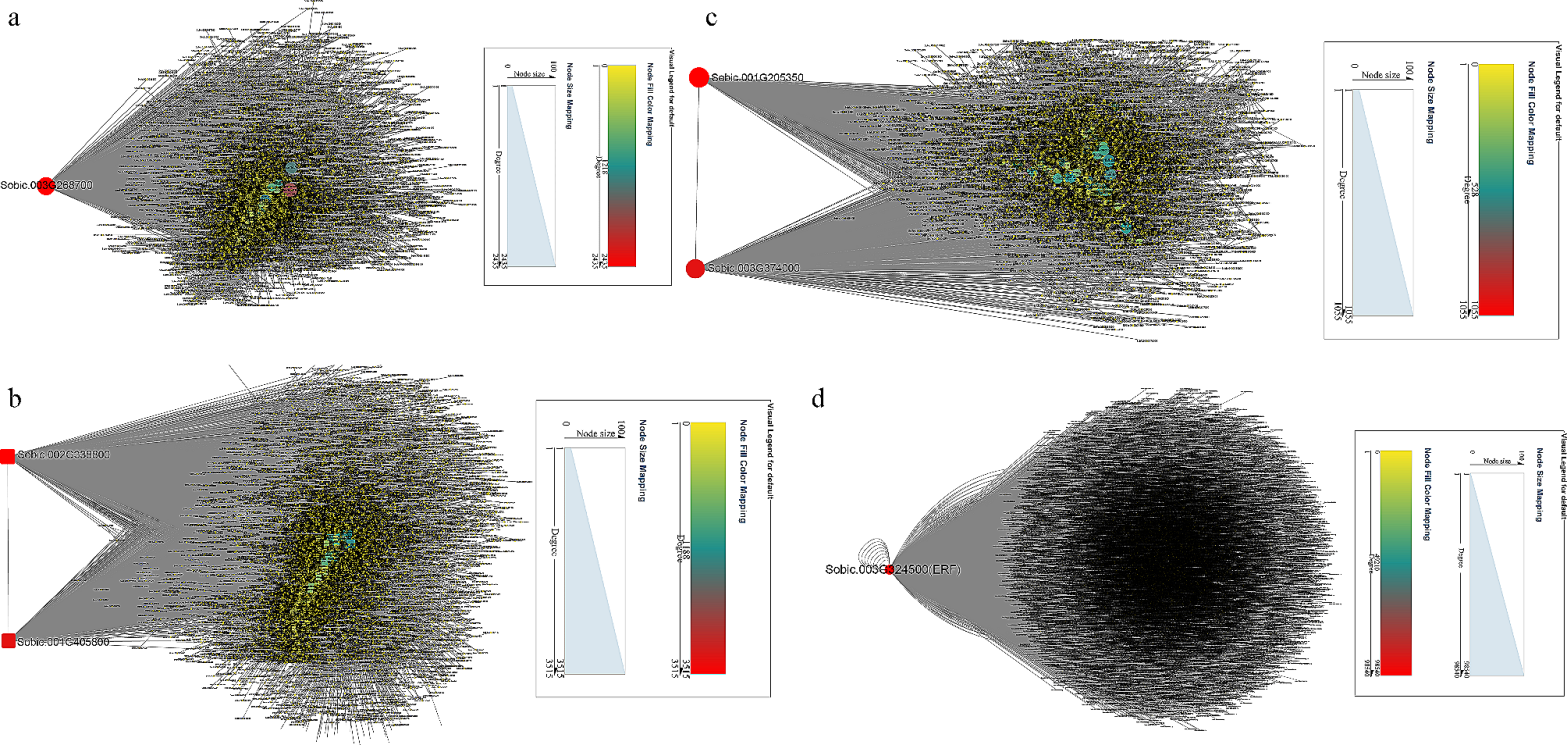
Cytoscape representation of relationship of genes in ‘brown4’, ‘coral1’ and ‘navajowhite2’ modules. (**a**) Cytoscape representation of relationship of genes in ‘brown4’ module. (**b**) Cytoscape representation of relationship of genes in ‘coral1’ module. (**c**) Cytoscape representation of relationship of genes in ‘navajowhite2’ module. (**d**) Cytoscape representation of relationship of TFs in ‘brown4’, ‘coral1’ and ‘navajowhite2’ modules

Networks were established to analyze hub genes in the above modules. In ‘brown4’ module, *Sobic.003G268700* belonging to protein kinase superfamily was identified as the key gene (Fig. 5a). In the network, three genes (*Sobic.001G017100* responding to water stress, *Sobic.004G247300* and *Sobic.004G302000* involving in salt stress) possibly interacted with *Sobic.003G268700* (Fig. 5a). *Sobic.002G338800* and *Sobic.001G405800* were determined in response to drought in ‘coral1’ module (Fig. 5b). The water stress-related genes *Sobic.001G017100* and *Sobic.003G271800*, and salt stress-related genes *Sobic.001G509800*, *Sobic.002G115200*, *Sobic.002G326800*, *Sobic.002G327700*, *Sobic.004G247300*, *Sobic.010G041700* and *Sobic.006G161200* shared interaction with the hub genes (Fig. 5b). There were two key genes (*Sobic.001G205350* and *Sobic.003G374000*) in ‘navajowhite2’ module; And two genes (*Sobic.001G017100* and *Sobic.003G271800*) which responded to water, and five genes (*Sobic.001G509800*, *Sobic.002G326800*, *Sobic.003G188000*, *Sobic.010G041700* and *Sobic.006G161200*) which referred to salt stress showed high correlation with the two hub genes (Fig. 5c). Considering potential roles of TFs in the response of sorghum to drought, Sobic.003G324500 (a ERF) was likely to be the most important gene in the above three modules based on TFs-genes network (Fig. 5d). And two water related genes (*Sobic.003G271800* and *Sobic.001G017100*), and several salt related genes (*Sobic.001G418200*, *Sobic.002G328800*, *Sobic.002G327700*, *Sobic.006G161200*, *Sobic.010G104400*, *Sobic.002G328900*, *Sobic.004G247300*, *Sobic.003G188000*, *Sobic.009G014700*, *Sobic.001G509800*, *Sobic.002G327201*, *Sobic.002G327300*, *Sobic.002G115200* and *Sobic.010G041700*) were predicted to the targets of ERF (Fig. 5d).

### Identification of DEGs in response to salt stress at root, leaf sheaths and leaf blades of sorghum

**Fig. 6:**
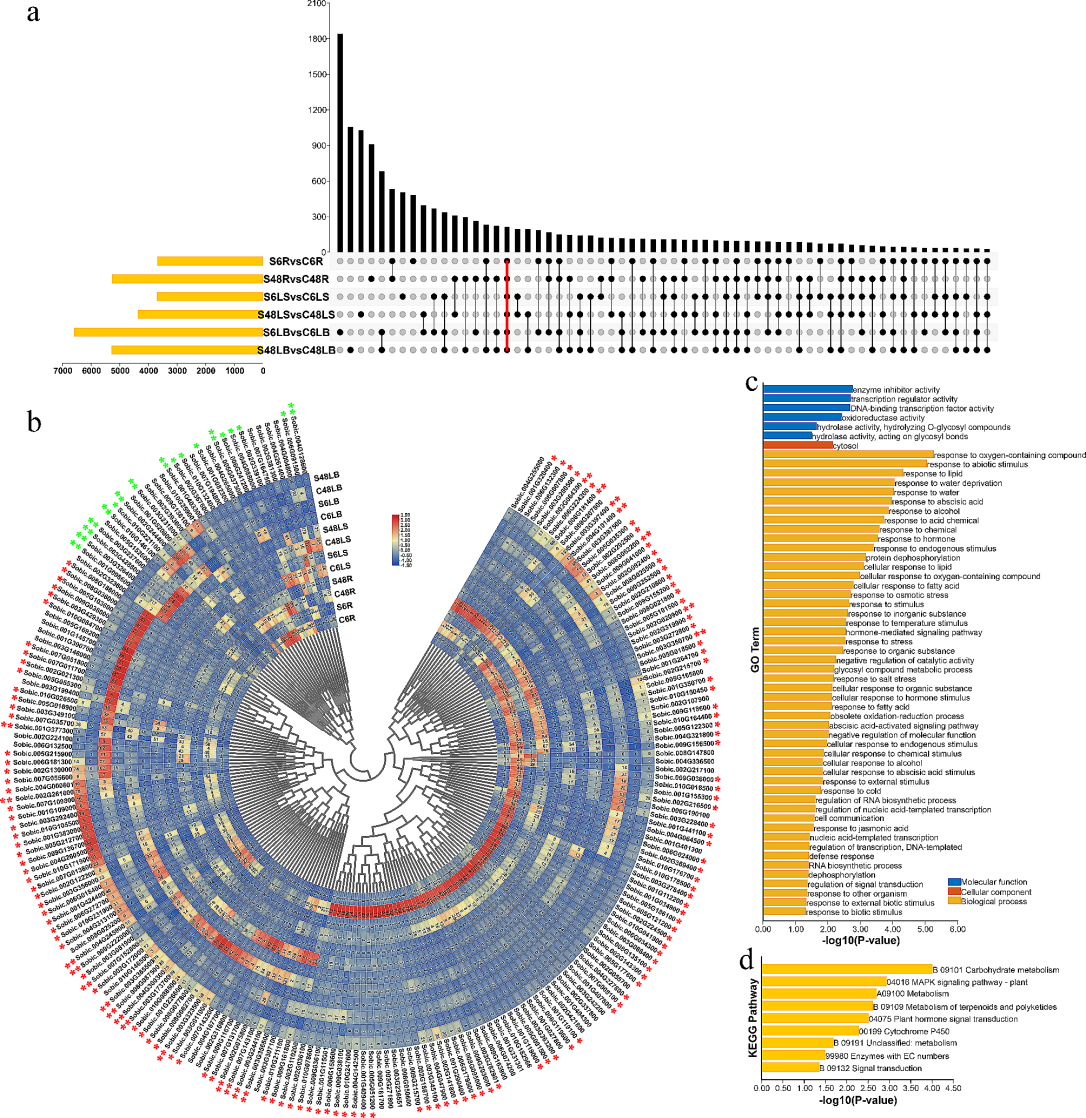
The DEGs between control and salt-treated sorghum seedlings at different tissues. (**a**) Venn diagram showing the common DEGs of the six pairwise comparisons. (**b**) Expression profile of the common DEGs in the six pairwise comparisons. Genes with high expression which repressed or induced by drought were labeled with green or red asterisk. (**c**) GO analysis of the common DEGs. (**d**) KEGG analysis of the common DEGs

The genes responding to salt were identified, there were 214 genes to be differently expressed at two time points in all tissues, and twenty-three of them were TFs (Fig. 6a; Table S18-S23). Many genes were commonly down-regulated or up-regulated after salt stress (Fig. 6b). A total of 18 genes were repressed by salt, 10 of them (*Sobic.004G128600*, *Sobic.005G037300*, *Sobic.001G403300*, *Sobic.003G231800*, *Sobic.002G244400*, *Sobic.010G146100*, *Sobic.003G237600*, *Sobic.003G428800*, *Sobic.003G326400* and *Sobic.001G098600*) showed relatively high expression, and *Sobic.003G428800* and *Sobic.003G231800* were two TFs (Fig. 6b). Over 100 genes were commonly induced by salt, and the expression of 21 genes were relatively high (Fig. 6b). These DEGs were related to abiotic stimulus, water, abscisic acid, osmotic stress and salt stress signals according to GO analysis (Fig. 6c). Based on KEGG analysis, these DEGs were enriched in carbohydrate metabolism and plant hormone signal transduction (Fig. 6d).

**Fig. 7:**
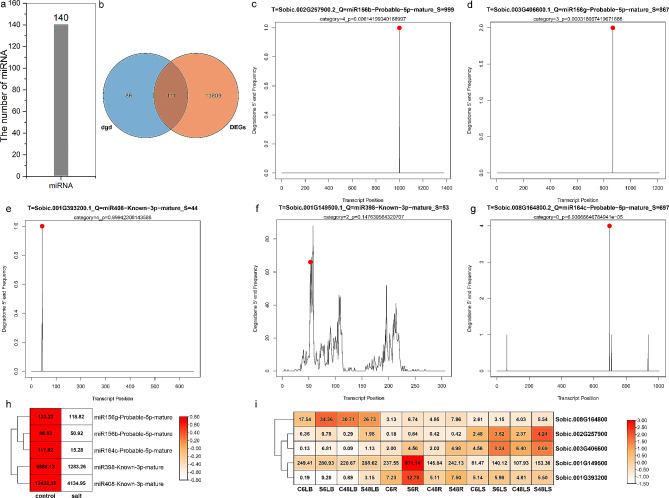
The analysis of miRNA and its target responding to salt. (**a**) The number of miRNAs identified in control and salt-treated sorghum. (**b**) Venn diagram showing the common genes between the targets of miRNAs identified by degradome sequencing and DEGs in response to salt stress. (**c**) Target plot (t-plot) for miR156b targets confirmed by degradome sequencing. (**d**) Target plot (t-plot) for miR156g targets confirmed by degradome sequencing. (**e**) Target plot (t-plot) for miR408 targets confirmed by degradome sequencing. (**f**) Target plot (t-plot) for miR398 targets confirmed by degradome sequencing. (**g**) Target plot (t-plot) for miR164c targets confirmed by degradome sequencing. (**h**) Expression analysis of miR5072 in response to drought. i Expression analysis of miR5072’ target (*Sobic.001G449600*) in response to drought

A total of 140 miRNAs may be involved in salt stress in sorghum (Fig. 7a). Using degradome sequencing, target genes of miRNAs were identified, and we found 111 of them were also DEGs (Fig. 7b). According to the target plots, miR156b, miR156g, miR408, miR398 and miR164c were predicted to bind to sites in the *Sobic.002G257900* (a SBP TF), *Sobic.003G406600* (a SBP TF), *Sobic.001G393200*, *Sobic.001G149500* and *Sobic.008G164800* (a NAC TF) mRNAs (Fig. 7c and g). The expression of these miRNAs were down-regulated by salt (Fig. 7h), while their target genes were generally induced by salt (Fig. 7i).

**Fig. 8:**
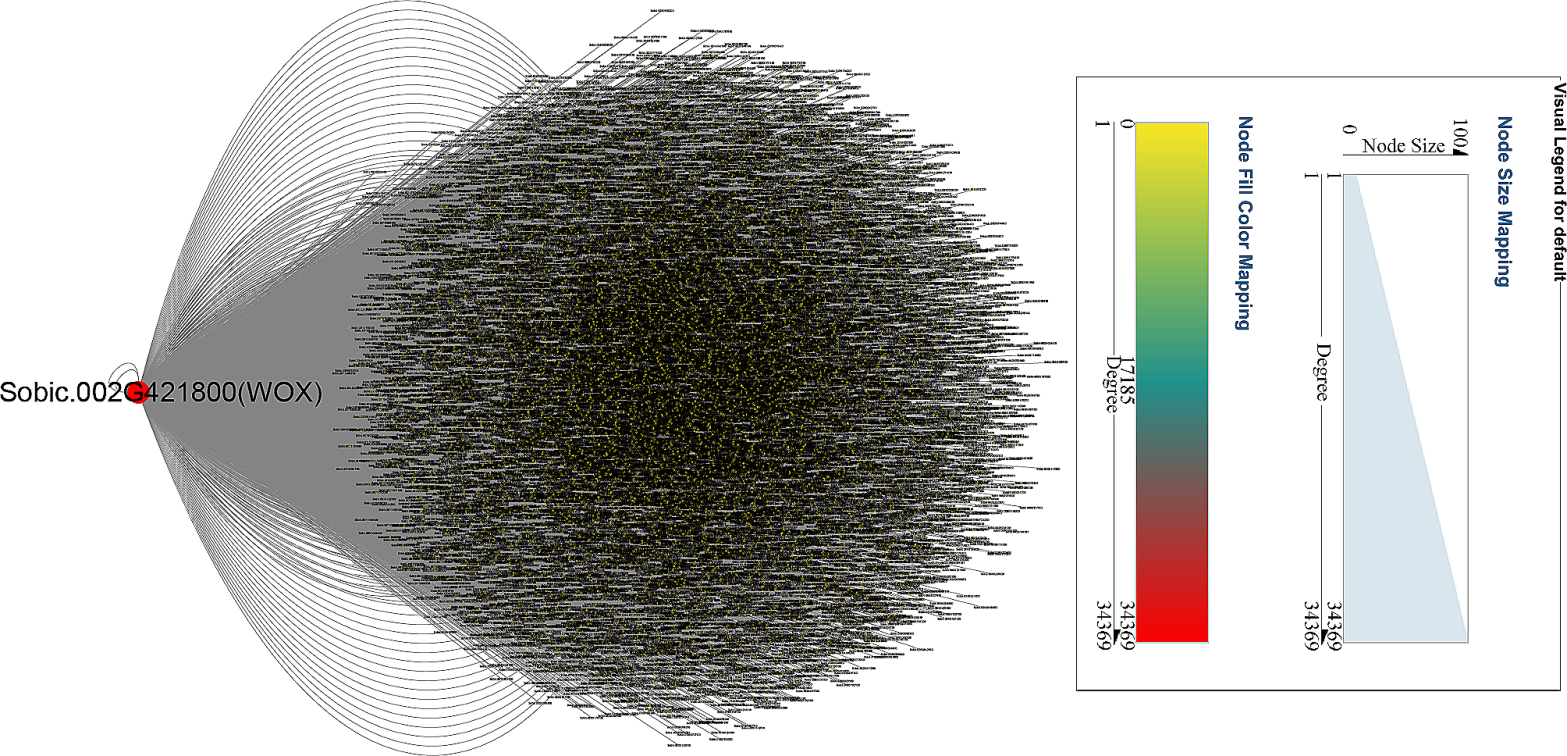
Regulatory network of TFs-mediated salt response in sorghum

Among the DEGs, *Sobic.002G421800*, a *WOX* gene, was likely to the most important gene (Fig. 8). Water-related gene *Sobic.003G271800*, and salt stress genes (*Sobic.003G193400*, *Sobic.010G104400*, *Sobic.004G247300*, *Sobic.002G326650*, *Sobic.002G326800*, *Sobic.009G004950*, *Sobic.002G409200*, *Sobic.003G188100*, *Sobic.009G014700*, *Sobic.002G327700*, *Sobic.002G327400* and *Sobic.004G302000*) were the potential targets of Sobic.002G421800 (WOX) TF.

### Identification of WGCNA modules and hub genes associated with salt stress

**Fig. 9:**
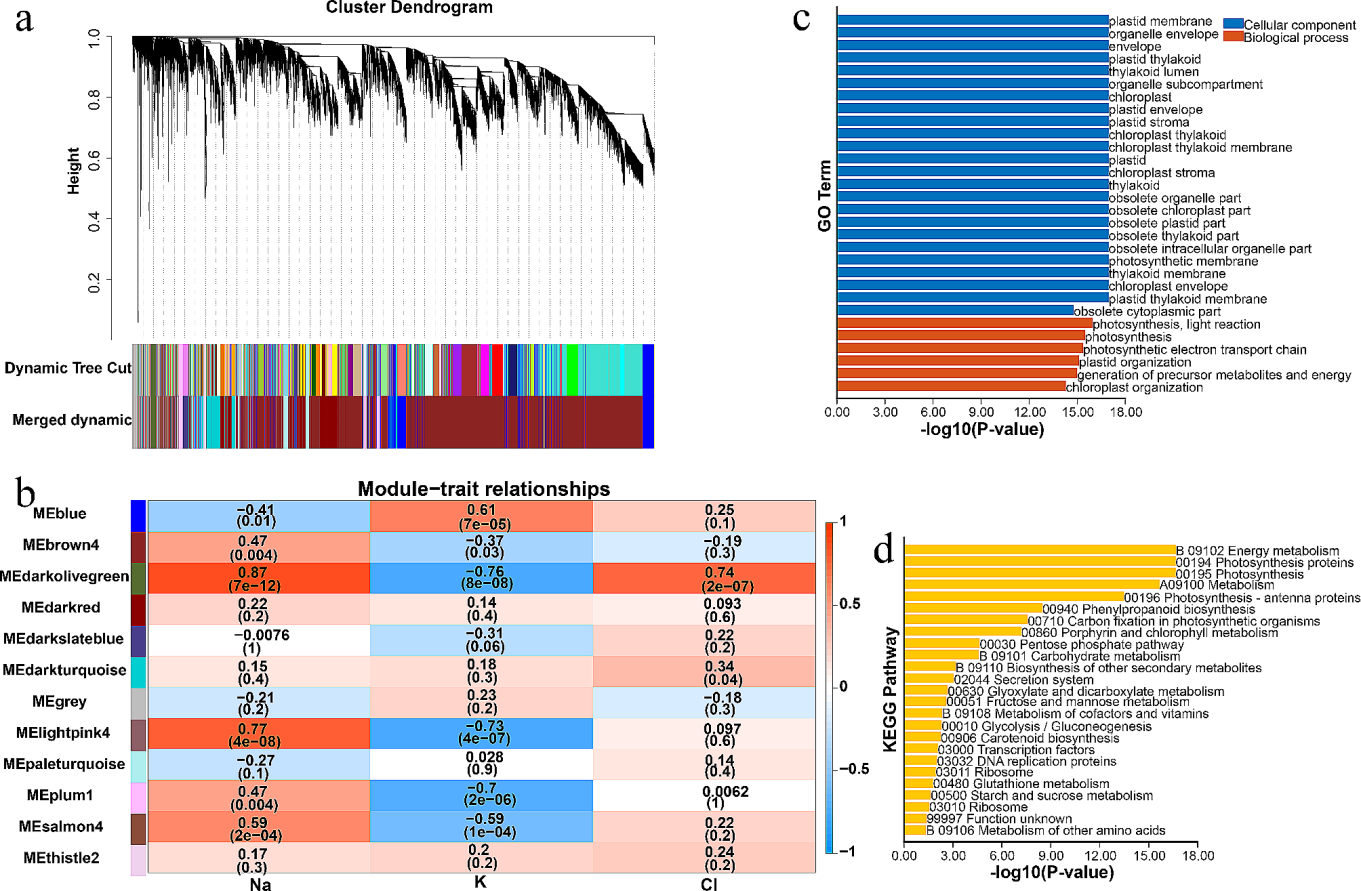
WGCNA of gene expression and Na^+^, K^+^ as well as Cl^−^ in sorghum uder salt stress. (**a**) Hierarchical clustering tree showing 12 modules of co-expressed genes by WGCNA. (**b**) The correlations between modules and sorghum growth. The number in each cell indicates the correlation coefficient (*r*), and the *P*-value (in parentheses) represents correlation significance (*P* < 0.05 indicated the significant correlation). (**c**) GO analysis of the genes in ‘darkolivegreen’, ‘lightpink4’ and ‘plum1’ modules. (**d**) KEGG analysis of the genes in ‘darkolivegreen’, ‘lightpink4’ and ‘plum1’ modules

Na^+^ and Cl^−^ were the dominant inorganic ions in salt toxicity. K^+^ and Na^+^ shared similar ion channels, excessive Na^+^ influx will reduce K^+^ in plants under salt stress [35]. Using WGCNA, 12 modules highly associated with Na^+^, Cl^−^ and K^+^ were identified (Fig. 9a). The ‘darkolivegreen’ module showed high correlation with Na^+^ (*r* = 0.87, *p* < 0.05), K^+^ (*r* = -0.76, *p* < 0.05) and Cl^−^ (*r* = 0. 74, *p* < 0.05), respectively; Na^+^ (*r* = 0.77, *p* < 0.05) and K^+^ (*r* = -0.73, *p* < 0.05) shared separately positive and negative correlation with ‘lightpink4’ module; And ‘plum1’ module shared negative relationship with K^+^ at *r* = -0.7 and *p* < 0.05 (Fig. 9b). As shown in Fig. 9c and d, the genes in above modules categorized into photosynthesis (photosynthesis, light reaction, photosynthesis, and photosynthetic election transport chain), energy metabolism, carbohydrate metabolism, biosynthesis of other secondary metabolites, glycolysis, carotenoid biosynthesis, fructose and mannose metabolism, and starch and sucrose metabolism.

**Fig. 10:**
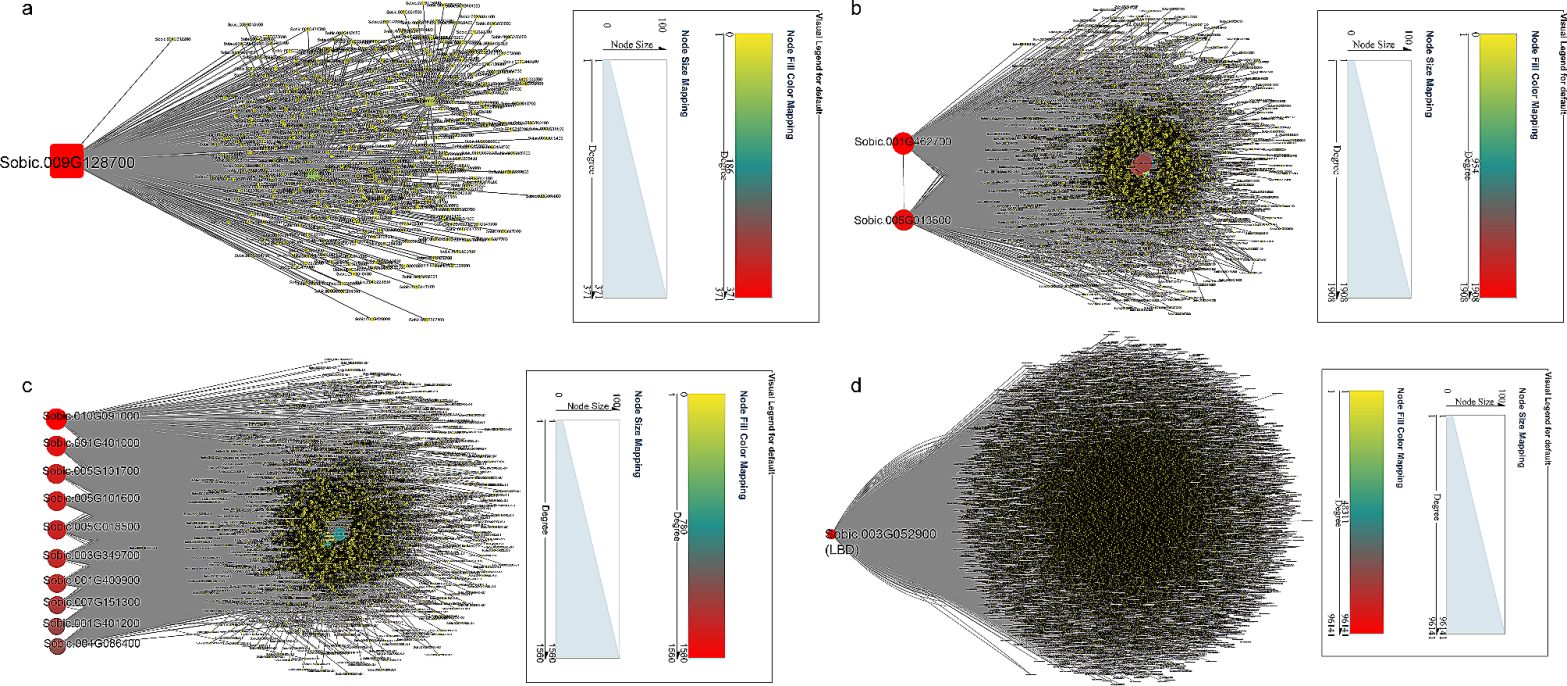
Cytoscape representation of relationship of genes in ‘darkolivegreen’, ‘lightpink4’ and ‘plum1’ modules. (**a**) Cytoscape representation of relationship of genes in ‘darkolivegreen’ module. (**b**) Cytoscape representation of relationship of genes in ‘lightpink4’ module. (**c**) Cytoscape representation of relationship of genes in ‘plum1’ module. (**d**) Cytoscape representation of relationship of TFs in ‘darkolivegreen’, ‘lightpink4’ and ‘plum1’ modules

In ‘darkolivegreen’ module, a co-expression network was constructed to identify hub gene. *Sobic.009G128700* were determined in response to Na^+^, K^+^ and Cl^−^ stresses, and a salt-related gene *Sobic.002G326800* and a water-related *Sobic.003G271800* were found to interact with the hub gene (Fig. 10a). *Sobic.001G462700* and *Sobic.005G013600* were the two most important genes in ‘lightpink4’ module, and *Sobic.003G271800* which responded to water deprivation may be under the control of the two hub genes (Fig. 10b). Ten genes (*Sobic.010G091000*, *Sobic.001G401000*, *Sobic.005G101700*, *Sobic.005G101600*, *Sobic.005G018500*, *Sobic.003G349700*, *Sobic.001G400900*, *Sobic.007G151300*, *Sobic.001G401200* and *Sobic.004G086400*) were hub genes in the ‘plum1’ module; *Sobic.001G509800*, *Sobic.002G327201* and *Sobic.002G327300* in response to salt stress, and *Sobic.003G271800* involving in water stress may be targets of the ten hub genes (Fig. 10c). In the above three modules, LBD (Sobic.003G052900) was the most important TF, and many salt-related genes (*Sobic.001G509800*, *Sobic.002G328800*, *Sobic.002G327201*, *Sobic.010G104400*, *Sobic.002G409200*, *Sobic.007G029000*, *Sobic.002G327300*, *Sobic.001G156600*, *Sobic.002G115200*, *Sobic.002G328900*, *Sobic.001G418200*, *Sobic.006G161200*, *Sobic.003G188000* and *Sobic.001G209600*) and three water stress-related genes (*Sobic.007G169000*, *Sobic.001G017100* and *Sobic.002G103900*) may be the possible targets of LBD (Fig. 10d).

### Venn analyses of genes mediating drought and salt stress adaption

**Fig. 11:**
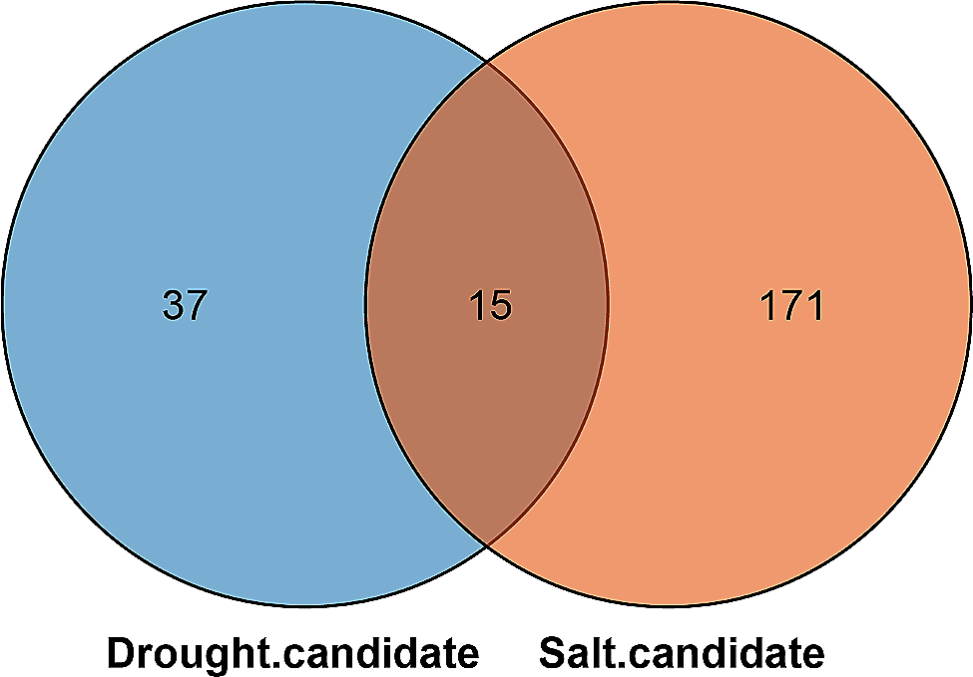
Venn diagram showing common candidate genes in response to drought and salt stresses

According to differential expression analysis and WGCNA, a Venn diagram was constructed to investigate the genes commonly respond to drought and salt stress. In total, 15 genes (*Sobic.008G062700*, *Sobic.004G142500*, *Sobic.004G047900*, *Sobic.001G034900*, *Sobic.001G155300*, *Sobic.010G084700*, *Sobic.006G018400*, *Sobic.008G087500*, *Sobic.009G161800*, *Sobic.003G244100*, *Sobic.005G055300*, *Sobic.001G424400*, *Sobic.004G300300*, *Sobic.001G226600* and *Sobic.010G247600*) were identified to involves in combined drought and salt stress, and two of them were TFs (Sobic.004G300300, HD-ZIP; Sobic.003G244100, bZIP) (Fig. 11 and Table S27).

## Discussion

Drought and salt are two of the most adverse abiotic stresses for plant growth and development, and they will affect crop yield and quality. The understanding in molecular mechanism of sorghum in response to drought and salt stress has made progress. However, information on systematic TFs identification, miRNAs-genes regulatory modules, and combined drought and stress adaption remain limited in sorghum. In this study, TFs were systematically characterized for their essential functions in directing interpretation of the genome and gene expression in sorghum [36]. The conserved domains and synteny of TFs were further analyzed. MiRNA and their target genes in response to drought and salt were identified. In addition, the gene expression profiles in response to drought and salt stress were identified through differential expression analysis and TF-gene network and WGCNA.

### Comparison of TFs between sorghum and model plants

There were more SbTFs and OsTFs compared with AtTFs (Figure S1). According to synteny analysis, more orthologous TFs were identified in *Arabidopsis thaliana* (503 pairs) than sorghum (447 pairs) and rice (400 pairs) (Figure S1). Sorghum and rice have been reported to undergo whole-genome duplication [37]. Therefore, the expansion and evolution of TFs in sorghum and rice may be caused by whole-genome duplications, not segmental duplications.

Duplicated blocks of sorghum-*Arabidopsis* and sorghum-rice were also identified, and respectively yielding 300 and 2010 TF pairs based on synteny analysis (Figure S1; Table S5 and S6). The sorghum TFs in pairs are likely to originate from common ancestors with the *Arabidopsis* and rice ones, indicating their similar functions with the corresponding model plants ones. We may predict the roles of sorghum TFs based on the *Arabidopsis* and rice ones, while these comparisons need to be verified in further experiments.

Gene function is closely associated with conserved domains [38]. With several exceptions, the domains in the TFs were typical among sorghum, *Arabidopsis* and rice (Table S1), suggesting that they may have conserved functions. However, the unique domains implied new gene functions and should be paid greater attention.

### The genes sharing key roles in the drought and salt tolerance of sorghum

In this study, to explore their functions, the genes expression patterns were determined under drought and salt stresses. A total of 47 common DEGs were found at drought-resistant and drought-sensitive sorghum genotypes (Fig. 1a), and they were involved in abiotic stress and energy metabolism (Fig. 1c and d). Among them, 41 DEGs were commonly induced by drought, and 18 of 41 DEGs shared high expression level in samples (Fig. 1b). MiR5072 and its target gene *Sobic.001G449600* may help examine the underlying mechanisms of drought resistance in sorghum using an integrated analysis of mRNA-seq, small RNA-seq and degradome (Fig. 2). *Sobic.008G050600* (*ERF*), *Sobic.007G077100* (*ERF*), *Sobic.003G324400* (*ERF*) and *Sobic.003G033500* (*Dof*) may play essential roles in drought stress response based on TF-DEGs network (Fig. 3). Using WGCNA, genes with similar expression patterns, and the relationship between modules and specific traits or phenotypes were clustered across multiple samples [39]. And WGCNA is widely used to identify the association between phenotypic traits and genes. Six hub genes, including a ERF TF, were identified in response to drought stress; And water stress as well as salt stress-related genes were the potential targets of hub genes (Fig. 5). Totally, 25 candidate genes in response to drought stress were found, and future studies should pay attention to these genes.

There were 214 common DEGs in response to salt stress based on GO and KEGG enrichment analysis (Fig. 6a, c and d). Among them, 18 and 148 genes were down-regulated or up-regulated by salt at all samples, and 31 genes (i.e., *Sobic.004G128600*, *Sobic.005G037300*, *Sobic.003G064300, Sobic.006G181400* and do on) with higher expression may have relatively important functions (Fig. 6b). Five miRNAs and their target genes may play essential roles in regulating sorghum salt resistance using an integrated analysis of mRNA-seq, small RNA-seq and degradome (Fig. 7). In TF-DEGs network, a WOX TF (Sobic.002G421800) was the hub gene and predicted to interact with water- and salt-related genes (Fig. 8). In three WGCNA modules sharing high correlation with salt, 14 hub genes, including a LBD TF, were identified (Fig. 10). Several genes responding to water deprivation and salt stress were likely to interact with core genes, suggesting that these core genes may take part in salt stress adaption by interacting with these genes. And the potential functions of these key genes should be focused in future studies.

Fifteen genes were identified as key genes in the adaption of sorghum to combined drought and salt stresses by differently expression analysis, TF-DEGs network analysis and WGCNA (Fig. 11). Considering TFs’ important biological functions, HD-ZIP (Sobic.004G300300) and bZIP (Sobic.003G244100) should be the most critical candidate genes for breeding drought-tolerant and salt-tolerant sorghum.

## Conclusions

In general, TFs in sorghum were systematically identified. Their chromosomal locations, conserved domains and syntenic relationships were characterized. Their responding to drought and salt were investigated through differential expression analysis, TF-DEGs network and WGCNA. Over than 15 genes, especially *HD-ZIP* (*Sobic.004G300300*) and *bZIP* (*Sobic.003G244100*), were identified as potential hub genes for improving the adaption of drought and salt. The functions of these genes should be validated experimentally in future.

## Methods

### TF identification, conserved domains, chromosomal location, and synteny

The TFs protein sequences of sorghum, *Arabidopsis thaliana* and rice were downloaded from Plant Transcription Factor Database (https://planttfdb.gao-lab.org/). Using the Batch Web CD-Search Tool (https://www.ncbi.nlm.nih.gov/Structure/bwrpsb/bwrpsb.cgi), the conserved domains in TFs were confirmed. Gene density were calculated with gene structure annotation (gff3) file, and visualized using “Advanced Circos” in TBtools. “One Step MCScanX” in TBtools was used to analyze TF duplication events with genome sequences and gff3 files. Gene pairs in TFs were identified with “File Merge for MCScanX” in TBtools. The Ka/Ks values of TF pairs were calculated with their coding sequences (CDS) using “Simple Ka/Ks Calculator (NG)” in TBtools.

### Transcriptome and sRNA analysis

The raw data of transcriptome (mRNA-seq), small RNA-seq and degradome were downloaded from NCBI database (https://www.ncbi.nlm.nih.gov/sra/) using accession numbers GSE157523, GSE157521, PRJNA977880, PRJNA585370 and PRJNA285718 [20, 22, 23]. Using fastp software (v0.20.1), the overall sequencing quality of these raw reads was evaluated, and low-quality reads were removed. With Hisat2 (v2.1.0) and SAMtools (v1.6) software, high-quality reads were aligned to sorghum reference genome sequences (https://phytozome-next.jgi.doe.gov/info/Sbicolor_v3_1_1). The Fragments Per Kilobase of exon model per Million mapped fragments (FPKM) values of high-confidence genes were calculated with stringtie (v1.3.3b) software. The DEGs were defined with *p* < 0.05, false-discovery rate (FDR) < 0.05 and |log2(fold-change)| ≥ 1 using the R package “edgeR”. MiRNAs identification was performed with sRNAminer software [11] according to sRNAminer Cookbook (https://www.yuque.com/u758713/at2327/drhlg8). CleaveLand4.pl was used to map the filtered degradome reads to sorghum cDNAs, and then identify the valid targets of miRNAs [40].

### Function enrichment analysis, WGCNA and TF-gene network construction

The gene expression profiles were visualized using “HeatMap” in TBtools [41]. GO and KEGG enrichments were performed with “GO Enrichment” and “KEGG Enrichment Analysis” in TBtools using background files which can be obtained from EggNOG-mapper (http://eggnog-mapper.embl.de/), and visualized with “Enrichment Bar Plot”. WGCNA was completed with high-quality genes using the R WGCNA package (v1.51). Significant module-trait relationships with target traits were determined by calculating modular trait gene values (|*r*| ≥ 0.69, and the *P*-value ≤ 0.01), and hub genes were the ones with high weight and degree in the significant modules [9, 38]. TF-gene network was constructed with “Plant TF Motifs Shift” and “Fimo: Binding Motif Scan” plugins of TBtools. The sorghum TF binding pattern was built with the protein sequences of sorghum using “Plant TF Motifs Shift”, and the gene-gene interacted network was analyzed with “Fimo: Binding Motif Scan”. With Cytoscape (v3.8.2) software, gene co-expression network maps were visualized. Venn diagrams were visualized using “UpSet Plot (Up to Any Sets)”.

### Electronic supplementary material

Below is the link to the electronic supplementary material.

Supplementary Material 1

Supplementary Material 2

Supplementary Material 3

Supplementary Material 4

Supplementary Material 5

Supplementary Material 6

Supplementary Material 7

## Data Availability

The raw transcriptome sequencing data were submitted to the National Centre for Biotechnology Information (NCBI) (http://www.ncbi.nlm.nih.gov/) under BioProject GSE157523, GSE157521, PRJNA977880, PRJNA585370 and PRJNA285718. The datasets used and/or analyzed in the current study are available from the corresponding author upon reasonable request

## Abbreviations

DEG: Differentially expressed gene
WGCNA: Weighted gene co-expression network analysis
TF: Transcription factor
Ka/Ks: Non-synonymous to synonymous substitution
Na^+^: Sodium
Cl^−^: Chlorine
K^+^: Potassium
