# Supplementary figures and images for: Revealing critical mechanisms in determining sorghum resistance to drought and salt using mRNA, small RNA and degradome sequencing
Source: BMC Plant Biol. 2024 Jun 13;24:547. doi: 10.1186/s12870-024-05230-1 (PMC11177356; doi:10.1186/s12870-024-05230-1)

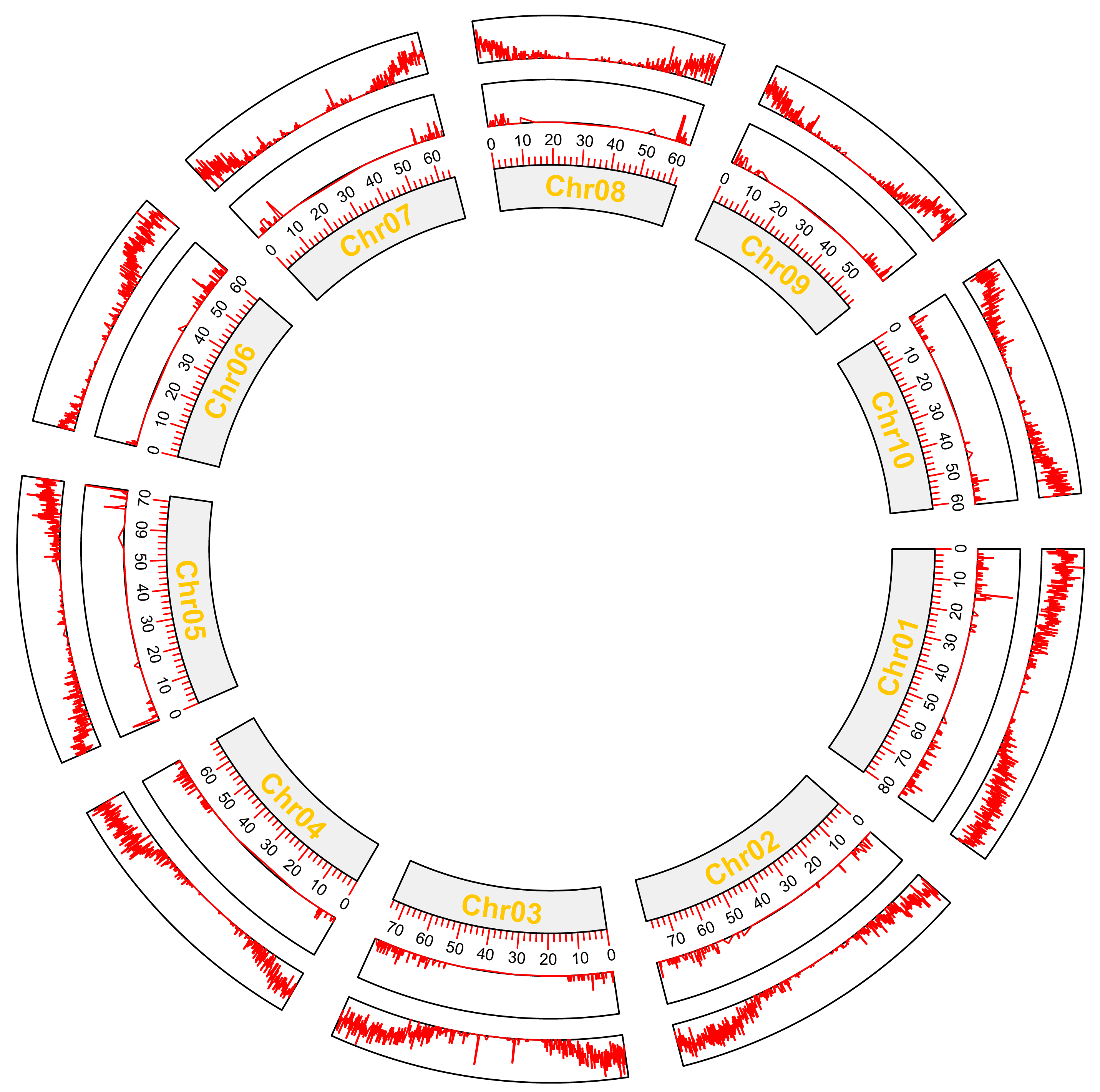

Supplement: Supplementary file 7 — Supplementary Material 7 [file 12870_2024_5230_MOESM7_ESM.tif]
